# Integrating Systemic Therapies into the Multimodality Treatment of Resectable Colorectal Liver Metastases

**DOI:** 10.1155/2018/4326082

**Published:** 2018-06-21

**Authors:** Omar Abdel-Rahman, Winson Y. Cheung

**Affiliations:** ^1^Clinical Oncology Department, Faculty of Medicine, Ain Shams University, Cairo, Egypt; ^2^Department of Oncology, University of Calgary, Tom Baker Cancer Centre, Calgary, AB, Canada

## Abstract

Colorectal carcinoma (CRC) is one of the most common cancers worldwide. A considerable proportion of CRC patients may present with metastatic disease either at upfront presentation (synchronous with the primary) or following diagnosis and treatment of the primary tumor (metachronous). Management of CRC liver metastases is a challenging endeavor which frequently necessitates proper assessment of patient- and disease-related factors. There is an opportunity within the management of CRC liver metastases to incorporate multiple treatment modalities (including surgery, other locoregional treatments, and systemic therapy). The current review aims to provide an updated overview on the optimal management strategy for CRC patients with liver metastases with a specific focus on the integration of systemic and/or locoregional treatments among patients with resectable or potentially resectable disease.

## 1. Introduction

Colorectal cancer (CRC) represents a major health problem worldwide and is considered one of the main causes of cancer-related morbidity and mortality [1]; CRC is expected to increase by 60% to more than 2.2 million new cases and 1.1 million deaths by 2030 [2].

A pragmatic, holistic approach to the management of CRC would consider patient-related, treatment-related, and disease-related variables [3]. Important patient-related variables include patient age, comorbidities, and their preferences and expectations. The most important disease-related factors to consider in the decision-making process are disease stage and biology [4, 5].

Patients with metastatic CRC are approached with a different treatment philosophy to the majority of other metastatic solid tumors. Because of the potential of cure among some of these patients, aggressive multimodality treatment strategies are frequently advocated. The most common site of metastases from CRC is the liver, and given the diversity of available systemic and locoregional treatments for CRC patients with liver metastases, establishing a reasonable and effective management approach for these patients may be a challenge for the treating physician [6].

Factors which could affect overall treatment decisions include extent of intrahepatic disease, presence/extent of extrahepatic disease, and timing of metastatic diagnosis in relation to the primary tumor (synchronous versus metachronous), patients' performance status, and disease biology [7].

The current review aims to provide an updated overview on the optimal management strategy for CRC patients with liver metastases with a specific focus on the integration of systemic and/or locoregional treatment among patients with resectable or potentially resectable disease.

### 1.1. Overview of the Management Strategy for Patients with CRC Liver Metastases

Overall, patients with CRC liver metastases may be classified into resectable, potentially resectable/convertible, and unresectable [8]. Different groups have published relevant guidelines for determining if and how patients should be classified into one of these three categories [9, 10]. In general, the key consideration within this classification scheme depends on the probability of achieving complete resection of all apparent disease with negative margins. Decisions about the proper classification of each patient as well as the optimal treatment approach should always be done in a multidisciplinary setting that incorporates input from all relevant stakeholders (including patient representatives). Ideally, such discussions should occur before the start of any active treatment for metastatic disease because treatment decisions may affect all subsequent aspects of the patient's care. In particular, the goals of care should be clear for all treating physicians at the start of treatment.

For patients with unresectable disease, the primary treatment modality is systemic therapy [11]. This might be administered as a single agent or in combination with a number of different agents. The choice of whether to embark on monotherapy or combination therapy is typically based on comorbidities, different toxicity profiles of each treatment, disease biology (particularly RAS mutational status), previous therapy exposure, and the degree of response to this previous therapy [12, 13]. If there is significant response to intensive first-line therapy, some patients may be additionally considered for maintenance treatment. This concept is appealing in the setting of unresectable disease given that the goal of care is palliative. As such, maintenance therapy offers disease control while minimizing the potential for significant toxicities which may have developed after 3 to 6 months of intensive first-line treatment [14].

In the management of patients who are considered unresectable, it is recommended that close follow-up of these patients be conducted so that appropriate and timely referrals to multidisciplinary teams can also be pursued, if needed. This is because some of these patients who are deemed initially as unresectable due to the proximity of critical structures may actually respond to initial therapy and become resectable or amenable to other locoregional interventions. The integration of locoregional interventions can improve the long-term control of the disease.

The use of epidermal growth factor receptor (EGFR) inhibitors (e.g., cetuximab or panitumumab) among RAS wild-type patients is particularly associated with high rates of conversion from unresectable into resectable disease. This was shown in multiple prospective studies as well as in a meta-analysis evaluating this endpoint [15].

On the other hand, the criteria upon which a patient with CRC liver metastases would be considered resectable have become more aggressive in recent years [16]. This was accompanied by a noticeable increase in the number of patients who are considered eligible for potential resection. This change was essentially related to a potential paradigm shift focusing on residual organ function after resection rather than the amount of disease that can be resected. Other factors which contributed to a relative increase in the number of patients considered resectable include the more frequent use of preoperative chemotherapy and the introduction of resection paired with local intraoperative ablation techniques. Generally speaking, in order for CRC liver metastases to be considered resectable, there should be no evidence of involvement of the hepatic artery, main portal vein, main bile duct, or para-aortic lymph nodes [17]. Moreover, there should be no evidence of unresectable extrahepatic metastases. Further, the primary tumor should be resected (either in the past or concurrently with the liver resection) and patients should be expected to retain adequate liver function post resection [18].

For patients with resectable or potentially resectable disease, various combinations and schedules for chemotherapy and locoregional treatments have been proposed in order to maximize the rate of R0 resections.

### 1.2. Systemic Therapy Options for Resectable/Potentially Resectable Disease

There is still a lack of consensus currently as to the optimal role and schedule of perioperative systemic therapy among patients with resectable CRC liver metastases. The potential advantages of perioperative therapy include treatment of possible micrometastatic disease, testing of the biological behavior of the tumor (patients with early progression on chemotherapy might be spared subsequent morbid surgery), and possible facilitation of the surgical resection. Overall, it is recommended that 6 months of perioperative fluoropyrimidine-based treatment be administered for patients with resectable CRC liver metastases [19].

In a phase III trial comparing FOLFOX perioperative chemotherapy versus surgery alone for patients with metastatic CRC, there was no observed significant difference in overall survival, despite the modest but significant progression-free survival benefit (3-year progression-free survival: 35.4% versus 28.1%, resp.; *P* = .058) [20]. Another meta-analysis of three randomized studies (*N* = 642) that compared surgery alone with surgery plus systemic therapy showed similar findings. The pooled analysis demonstrated a benefit of chemotherapy for progression-free survival (hazard ratio 0.75; 95% CI 0.62–0.91; *P* = .003), but there was no overall survival benefit (hazard ratio 0.743; 95% CI 0.527–1.045; *P* = .088) [21]. These findings underscore the importance for the multidisciplinary team to conduct close follow-up of these patients during chemotherapy in order to monitor for any untoward outcomes. Moreover, some systemic therapy agents may be associated with specific adverse surgical outcomes. For example, irinotecan-based regimens were linked to liver steatohepatitis, oxaliplatin-based regimens were linked to sinusoidal liver injury, and bevacizumab-based regimens were linked to higher risks of perioperative bleeding and thrombosis [22, 23].

Factors that inform the selection of appropriate chemotherapy regimens in the perioperative setting include previous chemotherapies used, the degree of response to these chemotherapies, and specific toxicities of each chemotherapy agent. The latter is clinically relevant, particularly when choosing therapies for patients with comorbidities. It is important to note that in the setting of resectable disease, there is little evidence that adding targeted treatments (anti-EGFR or antivascular endothelial growth factor) would improve the outcomes of resectable CRC liver metastases. Thus, most international guidelines do not consider this option for this clinical scenario. In line with this approach, the recent EPOC study showed that KRAS wild-type patients who received cetuximab plus FOLFOX as preoperative therapy had shorter median progression-free survival than patients who received chemotherapy alone (14.1 versus 20.5 months; hazard ratio 1.48; *P* = .030) [24].

The preoperative approach to patients with rectal carcinoma with liver metastases may differ than the approach used in colon cancer. Because of extra consideration for the need of locoregional control, neoadjuvant chemoradiotherapy may be offered as part of the perioperative treatment approach for rectal cancer patients [25].

### 1.3. Role of Chemotherapy during Surgery

Hyperthermic intraperitoneal chemotherapy (HIPEC) is a technique through which patients with either peritoneal carcinomatosis or high risk of peritoneal metastases receive heated, highly concentrated chemotherapy which is delivered directly to the abdomen during cytoreductive surgery [26, 27]. A retrospective study from the American Society of Peritoneal Surface Malignancies which examined overall survival in 539 patients with CRC with peritoneal carcinomatosis showed no difference between mitomycin C-based or oxaliplatin-based HIPEC treatment [28]. Thorough prospective assessment and standardization of this procedure are needed before routine implementation can be endorsed in clinical practice.

### 1.4. Role of Other Locoregional Treatments in addition to Surgery

For patients with no extrahepatic disease who are not eligible for complete surgical resection, other locoregional treatments might also be considered including radiofrequency ablation, hepatic arterial infusion chemotherapy, transarterial chemoembolization, or transarterial radioembolization.

Recent studies however have shown that the addition of radioembolization to first-line chemotherapy conferred no improvement in overall progression-free survival or response rate; however, there was an improvement in liver-specific progression-free survival [29].

## 2. Conclusions and Future Directions

The landscape of management of CRC patients with liver metastases has changed markedly over the past two decades with the introduction of new systemic therapy agents and locoregional treatments in addition to the adoption of different therapeutic paradigms with regard to the permissible extent of resection.

Patients with resectable CRC liver metastases could be treated with upfront resection to be followed by systemic therapy or by pre- and postoperative systemic therapy. In most cases, a total of six months of fluoropyrimidine-based treatment should be administered. Additional multidisciplinary discussions might include the timing of resection of primary (if present) versus the timing of resection of liver metastases as well as the timing of resection of extrahepatic resectable metastases (e.g., lung metastases).

A number of other agents have been recently approved for refractory/metastatic CRC (including aflibercept, regorafenib, and TAS-01). The possible role of these additional agents in the management of resectable CRC liver metastases needs further prospective evaluation. Likewise, immune checkpoint inhibitors have been approved recently for refractory/metastatic CRC with high MSI expression. It is as yet unclear how immune-oncology drugs should be incorporated into the perioperative setting. Other promising agents which might play a role in the personalized management of CRC liver metastases in the future include BRAF inhibitors (e.g., combination trametinib/dabrafenib) for BRAF mutant CRC or dual anti-Her2 therapy (trastuzumab and lapatinib) for HER2-positive disease [30].

Because of the potentially curative nature of some subsets of CRC liver metastastic patients, management of these patients should always be conducted within the setting of a coordinated multidisciplinary team. Integration of all available locoregional and systemic therapies should improve the outlook of these patients and provide reasonable long-term disease control.

## Additional Points

*Data*. The literature-based data supporting this review are from previously reported studies and datasets, which have been cited.
